# Molecular Signatures Reveal Circadian Clocks May Orchestrate the Homeorhetic Response to Lactation

**DOI:** 10.1371/journal.pone.0007395

**Published:** 2009-10-09

**Authors:** Theresa Casey, Osman Patel, Karl Dykema, Heather Dover, Kyle Furge, Karen Plaut

**Affiliations:** 1 Department of Animal Science, Michigan State University, East Lansing, Michigan, United States of America; 2 Department of Biology, Grand Valley State University, Allendale, Michigan, United States of America; 3 Van Andel Research Institute, Grand Rapids, Michigan, United States of America; Pennsylvania State University, United States of America

## Abstract

Genes associated with lactation evolved more slowly than other genes in the mammalian genome. Higher conservation of milk and mammary genes suggest that species variation in milk composition is due in part to the environment and that we must look deeper into the genome for regulation of lactation. At the onset of lactation, metabolic changes are coordinated among multiple tissues through the endocrine system to accommodate the increased demand for nutrients and energy while allowing the animal to remain in homeostasis. This process is known as homeorhesis. Homeorhetic adaptation to lactation has been extensively described; however how these adaptations are orchestrated among multiple tissues remains elusive. To develop a clearer picture of how gene expression is coordinated across multiple tissues during the pregnancy to lactation transition, total RNA was isolated from mammary, liver and adipose tissues collected from rat dams (n = 5) on day 20 of pregnancy and day 1 of lactation, and gene expression was measured using Affymetrix GeneChips. Two types of gene expression analysis were performed. Genes that were differentially expressed between days within a tissue were identified with linear regression, and univariate regression was used to identify genes commonly up-regulated and down-regulated across all tissues. Gene set enrichment analysis showed genes commonly up regulated among the three tissues enriched gene ontologies *primary metabolic processes*, *macromolecular complex assembly* and *negative regulation of apoptosis* ontologies. Genes enriched in *transcription regulator activity* showed the common up regulation of 2 core molecular clock genes, ARNTL and CLOCK. Commonly down regulated genes enriched *Rhythmic process* and included: NR1D1, DBP, BHLHB2, OPN4, and HTR7, which regulate intracellular circadian rhythms. Changes in mammary, liver and adipose transcriptomes at the onset of lactation illustrate the complexity of homeorhetic adaptations and suggest that these changes are coordinated through molecular clocks.

## Introduction

Taxonomic variation in milk composition is extensive, and is driven by neonatal requirements as well as life history and reproductive strategies of the dam. Maternal substrate demands of lactation are either met by increased dietary intake or by mobilization of nutrients stored in tissues [1], [2]. Recently, several high impact studies showed that although gene duplication and genomic rearrangement contribute to differences in the milk proteins among species, milk and mammary genes are more highly conserved than other genes in the mammalian genome [3], [4]. These findings suggest that we must look more deeply into the genome for the regulation of milk production to explain most of the species-specificity in milk composition.

Lactation is part of the reproductive process in mammals and is the most metabolically stressful period of an adult female's life [5]–[8]. In order for a dam to initiate lactation, her metabolic and hormonal milieu must be synchronized among multiple organs and organ systems so that nutrients are sent to the mammary gland for milk synthesis after birth [5], [6], [9]–[11]. This synchronized regulation is often referred to as homeorhesis, “coordinated changes in metabolism of body tissues necessary to support a (dominant) physiological process” [5], [6]. The central nervous system coordinates homeorhetic adaptations in the mother through the endocrine system. During pregnancy and at the onset of lactation there are dramatic changes in circulating levels of reproductive and metabolic hormones including estrogen, progesterone, placental lactogen, prolactin, leptin and cortisol [12]–[16]. Hormones stimulate metabolic changes in multiple organs so that nutrients and energy can be diverted to the fetus to support growth during pregnancy and then to the mammary gland to support milk synthesis at the initiation of lactation [17]–[19].

Previous work in our lab [20]–[25] has focused on describing the homeorhetic response to lactation, and we have developed a comprehensive data set that describes metabolic and physiological changes in the rat dam during the periparturient period. However there is little to no data that indicate how these changes are coordinated and how the central nervous system acts to mediate this response. The objective of our study was to try to determine a putative mechanism of how gene expression is coordinated across multiple tissues during the pregnancy to lactation transition. We describe changes in molecular signatures during the transition from pregnancy to lactation in mammary, liver and adipose using microarrays, and present our hypothesis based on these signatures that: homeorhetic adaptations to lactation are coordinated by circadian clocks and may account for some of the taxonomic variation in milk.

## Results and Discussion

### Coordinated changes in rate of lipid synthesis in mammary, liver and adipose during the transition from pregnancy to lactation

**Figure 1:**
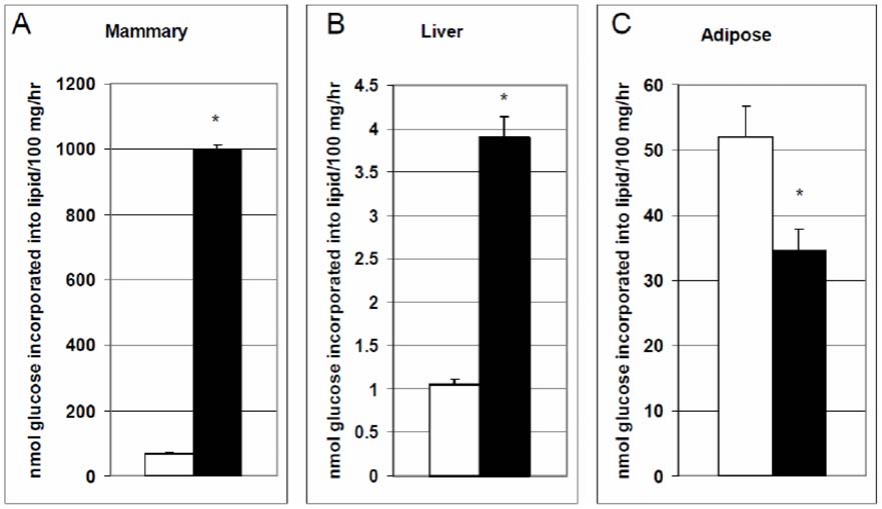
*In-vitro* rate of incorporation of glucose into lipids. The rate of glucose incorporation into lipids was measured in A) mammary, B) liver and C) adipose tissues on pregnancy day 20 (P20; white bars) and lactation day 1 (L1; black bars). Tissue slices were incubated in Krebs-Ringer Bicarbonate (KRB) buffer in the presence of 1 µCi/flask U-14C -glucose as a tracer. Rate of glucose incorporation into lipids expressed as nmoles glucose incorporated into lipids/100 mg tissue/hr. Values are expressed as mean±SE.

The *in-vitro* rate of incorporation of ^14^C-labeled glucose into lipids was used as an indicator of the *in-vivo* metabolic capacity of mammary, liver and adipose tissue on pregnancy day 20 (P20) and lactation day 1 (L1). Rate of lipid synthesis on P20 was low in mammary tissue, when the mammary gland was not synthesizing milk. With the onset of lactation there was approximately a 10-fold increase in the rate of lipid synthesis in the mammary gland (Figure 1A). This increase in lipid synthesis is needed to supply milk fats to the neonate.

Although the rate of lipid synthesis was relatively low in liver, there was a significant increase during the transition from late pregnancy to lactation (Figure 1B; P<0.005). We verified these results using acetate as a substrate, and found that the response to the onset of lactation was similar but the rate of lipid synthesis using acetate as a substrate was greater in liver (data not shown). The four-fold increase in lipid synthesis likely reflects the liver's role in providing lipids for synthesis of milk in the mammary gland. The decrease in rate of fat synthesis from P20 to L1 in adipose tissue (Figure 1C) indicates a decrease in its ability to store nutrients, thus indicating metabolic changes during this transition insure that nutrients are available for milk synthesis in the mammary gland.

### Coordinated changes in gene expression among multiple tissues during the transition from pregnancy to lactation

The orchestrated switch in lipogenesis from adipose tissue to the mammary gland is controlled by the hormonal environment which results in tissue-specific changes in the transcription and activity of enzymes that regulate lipogenesis [12], [14], [26]–[29]. However, there is limited information on the coordinated transcriptional regulation among the mammary, liver and adipose tissues during the transition from pregnancy to lactation.

In order to characterize the global gene expression patterns in liver, mammary and adipose tissues, total RNA was isolated from mammary, liver and adipose tissue from rat dams on P20 and L1 and gene expression was measured using Rat 230 2.0 Affymetrix GeneChips. Two types of gene expression analysis were performed. Linear regression was used to identify genes that were uniquely up and down-regulated in each tissue following the transition from P20 to L1 (i.e. mammary tissue on L1 versus mammary tissue on 20), and univariate regression was performed to identify individual genes that were commonly up-regulated and down-regulated across all the L1 tissues versus all the P20 tissues [30].

When nominal P-values were adjusted with false discovery rate, mammary tissue had by far the greatest number of statistically significant changes in gene expression during the transition from pregnancy to lactation (Table 1). Only 18 genes were significantly changed at the P<0.001 level in liver, and no genes were significant at this level in adipose tissue. The lack of large transcriptional changes in liver and adipose during this transition was not surprising. We expected moderate changes in gene expression relative to mammary, as the dam is already in a catabolic state in late pregnancy, which is enhanced in these two tissues at the onset of lactation to accommodate the increased energetic demands of milk synthesis [31]–[38]. Changes in metabolism in liver and adipose during this transition are thus likely to be subtle and may include regulation at the post-transcriptional level.

**Table 1:** No. genes differentially expressed between pregnancy and lactation in each tissue and common to all tissues.

	Common		Mammary		Liver		Adipose	
P-Value	adjusted	nominal	adjusted	nominal	Adjusted	nominal	adjusted	nominal
**0.0001**	12	334	1674	2255	7	73	0	21
**0.001**	68	1005	2506	3070	18	150	0	78
**0.01**	897	2766	3582	4223	62	428	8	321
**0.05**	3104	4854	4615	5393	112	1143	21	1064
**0.1**	4624	6069	5287	6019	178	1800	66	1851

There were 68 genes (P<0.001) commonly up- and down-regulated across all three tissues (Supplemental Table S1). Several of the genes commonly up regulated encode proteins involved in chaperone and stress response, actin cytoskeleton assembly, transcellular/intracellular trafficking as well as neural related signaling. The fact that a greater number of genes were significantly changed when examined for common regulation, was in part, due to the greater number of arrays compared (*n* = 15 across tissues versus *n* = 5 within tissues).

### Parametric gene set enrichment analysis (PGSEA) was used to explore coordinated changes in gene expression in mammary and liver and adipose tissue

**Figure 2:**
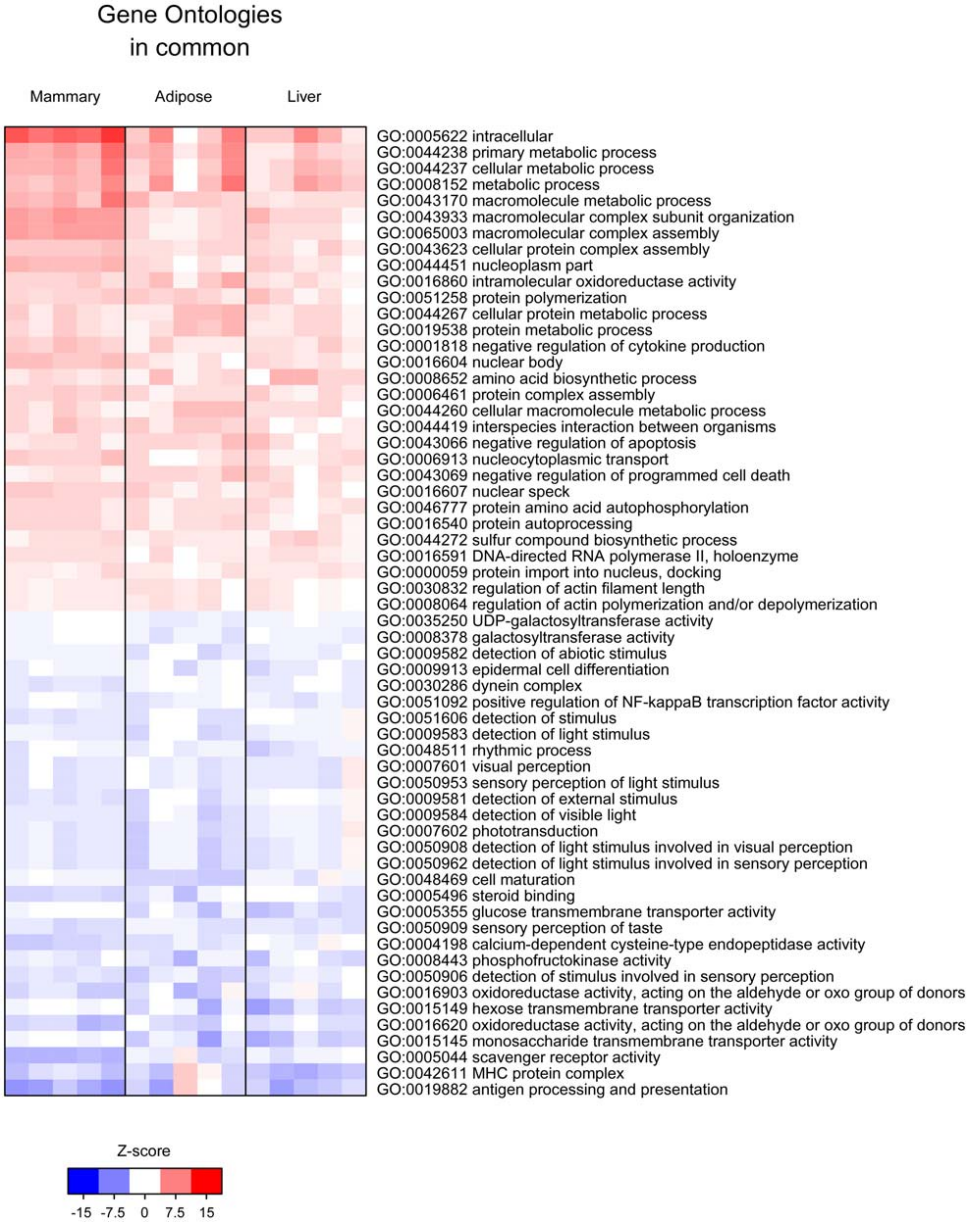
Gene ontology (GO) gene sets enriched with genes commonly up regulated or down regulated genes across all tissues (mammary, liver and adipose) during the transition from pregnancy to lactation. Each column represents data from an individual lactating (L1) rat compared to the average of the 5 pregnant rats (P20). For each L1 rat comparison, enrichment scores for each pathway were calculated and the pathways that were most consistently deregulated across the tissues were identified and the results plotted as a heat map [30]. Red indicates an enrichment of up regulated genes in the ontology/pathway and blue indicates enrichment of down regulated genes in the ontology/pathway during the P20 to L1 transition. Ontologies/Pathways were only scored if they had at least 10 genes represented in each category.

**Figure 3:**
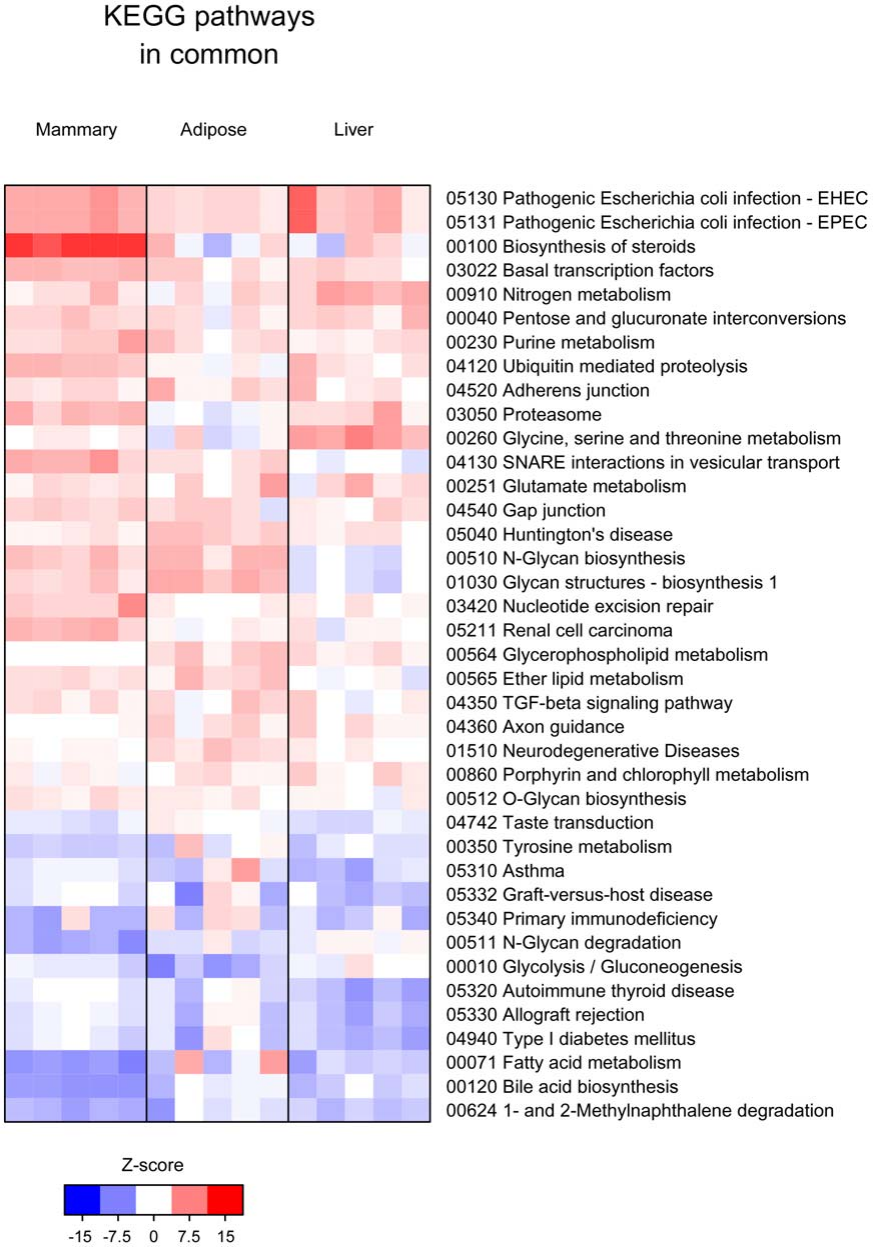
KEGG Pathway gene sets enriched with genes commonly up regulated or down regulated genes across all tissues (mammary, liver and adipose) during the transition from pregnancy to lactation. Each column represents data from an individual lactating (L1) rat compared to the average of the 5 pregnant rats (P20). For each L1 rat comparison, enrichment scores for each pathway were calculated and the pathways that were most consistently deregulated across the tissues were identified and the results plotted as a heat map [30]. Red indicates an enrichment of up regulated genes in the ontology/pathway and blue indicates enrichment of down regulated genes in the ontology/pathway during the P20 to L1 transition. Ontologies/Pathways were only scored if they had at least 10 genes represented in each category.

Gene set enrichment analysis approaches are designed to detect modest but coordinated changes in the expression of groups of functionally related genes [39], [40]. For our analysis, genes were first grouped into sets based on Gene Ontologies (GO) and KEGG pathways, pathway enrichment scores were computed for each gene set using the parametric gene set enrichment approach, and gene sets that showed transcriptional differences between L1 and P20 tissues were identified. The most significant GO and KEGG pathways enriched with genes up or down regulated in mammary, liver and adipose are illustrated in Supplemental Figures S1, S2 and S3, respectively; ontologies and pathways that were significantly enriched with genes commonly up- or down-regulated across all three tissues are shown in Figures 2 and 3. Although gene set enrichment analysis provides a more systemic view of the gene expression data, a disadvantage of gene set enrichment approach is that pathways as a whole can be difficult to validate experimentally. Therefore, we highlighted some individual genes found within the genes sets to give the reader insight into the static nature of GO and KEGG pathway terms alone. Examination of individual genes also allowed for further biological interpretation of gene sets and hypotheses development. Adjusted and nominal P-values for genes within sets discussed in the manuscript are supplied in supplemental files (Supplemental Tables S1-S10).

### Changes in the molecular signature of the mammary gland during the transition from pregnancy to lactation

Gene sets enriched with genes up regulated during the pregnancy to lactation transition in the mammary gland reflect the turning on of secretory processes in this tissue. These gene sets included the GO: *endomembrane system, endoplasmic reticulum, transport, establishment of protein localization* as well as the KEGG pathway *SNARE interactions in vesicular transport* (Supplemental Figure S1A). Up regulated genes were also enriched in the *ubquitin related proteolysis* and *proteasome* KEGG pathways; enrichment of genes in these sets reflect the important role of ubiquitination pathways in the hormonal regulation of secretory activation in the mammary gland [41]–[43] and suggest that post-translation regulation is also needed for initiation of lactation. The *mTor signaling pathway* was enriched with genes up regulated during the transition from pregnancy to lactation (Supplemental Figure S1B). mTOR plays a central role in signaling caused by nutrients and mitogens. mTOR positively regulates translation and ribosome biogenesis while negatively controlling autophagy, and is believed to set protein synthetic rates as a function of the availability of translational precursors [44].

Gene sets enriched with genes down regulated in the mammary gland during the pregnancy to lactation transition included GO: *autophagy* and *aminopeptidase activity*, as well as the KEGG pathways: *N-glycan degradation, ABC transporter activity* and *PPAR signaling* (Supplemental Figure S1). Genes enriched in the PPAR signaling pathway encode proteins involved in lipid transport, lipid metabolism, particularly beta-oxidation and 2 nuclear receptors: RXR and the orphan receptor NR1H3 (aka LXR-alpha) that activates RXR (see Supplemental Table S2 for genes in this pathway). This molecular signature is consistent with the function and activity of the mammary gland during lactation, i.e. a down regulation of catabolic activity and sequestering of substrates to be used for milk synthesis through the down regulation of membrane transporters. The enrichment of down regulated genes in GO–*regulation of cell shape/cell morphogenesis, Rab/Ras GTPase binding*, and *activation of JNK activity*–is indicative of completion of mammary differentiation at the onset of lactation.

### Changes in the molecular signature of the liver during the transition from pregnancy to lactation

Gene sets enriched with genes up regulated in liver were related to P450 pathways which catalyze many reactions involved in drug metabolism and synthesis of cholesterol, steroids and other lipids (Supplemental Figure S2). Genes within these genes sets were found to encode proteins involved in synthesis of estrogen and retinoic acid, conversion of cholesterol to bile acids, or function within the arachidonic acid pathway. Enrichment of up regulated genes in *glutathione transferase activity* may be indicative of the increase in metabolic activity of liver during the pregnancy to lactation transition. Enzymes with this activity participate in the detoxification of reactive electrophilic compounds that are often by-products of metabolism.

The *adipocytokine signaling pathway* was enriched with 8 genes (P<0.05, nominal p-value; Supplemental Table S3) down regulated in liver at the onset of lactation. Genes within this set included: LEPR (the leptin receptor), PPARGC1A, CPT2, CPT1A, and PRKAB1. PPARGC1A encodes a transcriptional coactivator that regulates genes involved in energy metabolism. CPT2 stimulates beta oxidation of fatty acids, and CPT1A encodes a key enzyme involved in carnitine-dependent transport of long-chain fatty acids across the mitochondrial inner membrane and its deficiency results in a decreased rate of fatty acid beta-oxidation. PRKAB1 encodes a protein that positively regulates AMP-activated protein kinase (AMPK), an important energy-sensing enzyme that monitors cellular energy status.

Leptin plays a role in regulating food intake and adiposity centrally [45] but also acts peripherally to exert an antilipogenic, pro-oxidative action on its peripheral nonadipose target tissues, by lowering expression of lipogenic transcription factors, such as sterol regulatory element-binding protein (SREBP)-1c in liver and peroxisome proliferator-activated receptor (PPAR)-γ2 as well as lipogenic enzymes, including acetyl CoA carboxylase and fatty acid synthase (for review [46]). This transcriptional signature is likely a homeorhetic adaptation that reduces breakdown of fatty acids in the liver so that fats can be spared for milk synthesis in the mammary gland, and may be partly responsible for the 4-fold increase in the rate of lipogenesis we report for the liver (Figure 1).

The gene set *transmembrane receptor activity*, was enriched with 33 genes (P<0.05, nominal p-value; Supplemental Table S4; Supplemental Figure S2) down regulated in liver during the transition from pregnancy to lactation. Many of the genes within this set encoded proteins involved in feeding behavior, satiety and homeostasis, and included: LEPR, PRLR (prolactin receptor), INSRR (insulin-receptor related receptor), PPRY1 (receptor for neuropeptide Y and peptide YY), GNAT2 (a G-protein involved in transmission of visual impulses), GRPR (gastrin releasing peptide receptor), GPR50 (an orphan receptor that heterodimerizes with melatonin receptor), HTR7 (5-hydroxytryptamine (serotonin) receptor 7) CHRNA2 (a cholinergic receptor), HRH1 (a histamine receptor), and GFRA3 (receptor for neurotroph ARTN, artemin). Expression of many of these genes are classically associated with the central and enteric nervous system and regulate energy balance and feeding behavior [47], [48], thus providing clues to the endocrine and neuroendocrine responses that need to be investigated to fully understand the homeorhetic response to lactation.

### Changes in the molecular signature of the adipose tissue during the transition from pregnancy to lactation

Adipose tissue has traditionally been viewed as an inert energy storage tissue containing a fixed number of adipocytes, but now it is designated as a very dynamic endocrine organ with pleiotropic functions [49], [50]. Adipocytes secrete factors that play a central role in the regulation of energy balance, immunological responses and inflammation [49], [51]. The enrichment of the *complement and coagulation cascade* KEGG pathway and the GO *complement activation* and *activation of plasma proteins* (Supplemental Figure S3) during the transition from pregnancy to lactation indicate that both innate immunity and the complement system are up regulated, and suggests that an inflammatory response may be activated at the onset of lactation [52]. The enrichment of up regulated genes within sets related to intracellular transport, membrane trafficking, and secretion *endoplasmic reticulum, Golgi apparatus, melanosome* and *vesicle mediated transport* are likely due to the increased rate of lipolysis and transport of stored fats out of adipose tissue into circulation to supply energy and fats needed for milk synthesis.

GO enriched with genes down regulated during the pregnancy to lactation transition were overwhelmingly related to muscle contraction sacromere, myofibrils, and cytoskeleton. Twelve genes enriched the *contractile fiber* gene set (P<0.05, nominal P-value; Supplemental Table S5) and included: ACATA1 (actin alpha 1), MYH3 (myosin), and TNNT2 (troponin). Interestingly, insulin-induced translocation of glucose through GLUT4 protein is dependent on microtubules, and without microtubules glucose transport is highly diminished [53]–[55], suggesting that the decrease in expression of cytoskeletal genes in adipose tissue helps to spare glucose use by peripheral tissues. Interestingly, the transcriptome pattern revealed in adipose tissue during the transition from pregnancy to lactation showed a striking similarity to that observed with long-term caloric restriction. The molecular signature of adipose tissue between rats exposed to long-term caloric restriction and control rats revealed that 120 out of 345 differentially expressed genes were associated with metabolism (carbohydrate, lipid, amino acid and central aspects of energy metabolism) [56], [57], and the other 108 differentially expressed genes were classified as within ontology related to the cytoskeleton, ECM, inflammation and angiogenic activities [56], [57].

### Gene sets of commonly up and down regulated in mammary, liver and adipose tissue during the pregnancy to lactation transition

Not surprising, the majority of the gene sets enriched with genes commonly up regulated among all three tissues were related to metabolic processes (*primary metabolic process, macromolecular complex assembly, cellular protein metabolic process)* (Figure 2). Interestingly the most highly enriched KEGG pathways with commonly up regulated genes were *Pathogenic Escherichia coli infection – EHEC* and *Pathogenic Escherichia coli infection- EPEC*. Genes within these pathways were involved in the toll-like receptor pathway and adherens junctions. Gene sets enriched with commonly up regulated genes, also indicated apoptosis/programmed cell death was being inhibited during the transition from pregnancy to lactation in all three tissues.

It is interesting to point out that ATRN, attractin, was one of the most significantly commonly up regulated genes (Supplemental Table S1). Attractin is a low affinity receptor for agouti, and both of these molecules regulate pigmentation. Agouti is an antagonist for the melanocortin receptors, MC1R and MC4R [58], [59] [60]. Chronic antagonism of the cutaneous MC1-R by Agouti results in yellow fur and Agouti competition at the hypothalamic MC4-R results in obesity. Attractin (mahogony) knock out mice have increased basal metabolic rate and activity [61]. These data suggest that inhibition of melanocortin signaling through ATRN may be a major homeorhetic adaptation for lactation [61], [62].

In order to gain insight into what is stimulating these changes, we took a closer look at genes that were clustered into the gene ontology (GO: 0003700) *transcription factor activity* (Supplemental Table S6). There were 112 genes commonly up regulated among mammary, liver and adipose in this category. Many of these genes encoded proteins that functioned: to regulate metabolism (NR1I3, PTRF, MLX), as coactivators for nuclear receptors (NCOA1, NCOA3, NCOA4, MED13L, POU2F), or to transcriptionally regulate progression through the cell cycle (ARID4A, HELLS, MCM6). However, most interesting to us, was the common up regulation of 2 core molecular clock genes, ARNTL (aka BMAL1) and CLOCK as well as the up regulation of SREBF2. ARNTL and CLOCK gene products make up core clock elements that generate circadian rhythms. Heterodimers of ARNTL/CLOCK gene products activate transcription of numerous target genes that in turn show circadian patterns of expession either directly via E-box regulatory element in their promoter regions, or indirectly by other transcription factors whose expression is under clock control [63], [64]. SREBF2 encodes a sterol receptor binding protein transcription factor that activates enzymes important to de novo lipid synthesis.

There were 97 genes commonly down regulated among mammary, liver and adipose tissues that enriched the *transcription factor activity* GO gene set (Supplemental Table S7). Many of the products of these genes regulate developmental processes and included several classes of homeobox genes, the homeobox genes (HOXC9, HOXA5), sry (sex determining region) homeobox genes (SOX4, SOX10, SOX 15, SOX 21), and the Iroquois homeobox genes (IRX1, IRX3, IRX4, IRX5), a signature that indicates completion of differentiation at the onset lactation. There were also three genes within the transcription factor activity ontology cluster that were associated with the setting of the intracellular molecular clock and included: NR1D1, DBP, and BHLHB2. These three genes also enriched the GO *Rhythmic process* (GO:0048511; Figure 2; Supplemental Table S8) gene set that additionally included HTR7 and OPN4. HTR7 encodes a serotonin receptor (5-hydroxytryptamine receptor 7). Serotonin regulates tissue metabolism as well as entrains circadian rhythm phases [65]–[67]. OPN4 encodes a photoreceptor, melanopsin, required for regulation of circadian rhythm. It is intriguing that the expression of a retinal associated gene is regulated in non-ocular tissues, and suggests that another role may be attributed to melanopsin: regulator of peripheral tissue rhythms.

Other gene sets enriched with genes commonly down regulated among the three tissues were related to perception and transduction of external stimuli, light and taste, and included the GO sets, *Sensory perception of taste, Phototransduction*, and *Detection of stimulus* (Figure 2). There were 24 commonly down regulated genes (P<0.05 adjusted, within common genes; Supplemental Table S9) that enriched the gene set *Sensory perception of light stimulus*. Genes within this set encoded proteins classically known to receive, integrate and transmit light stimuli. Interestingly, a mutation of one of the genes in this set, BBS7, is associated with Bardet-Biedl syndrome. This syndrome is a genetically heterogeneous disorder characterized by severe pigmentary retinopathy and early onset obesity. Secondary features include diabetes mellitus, hypertension and congenital heart disease [68]. Mice with knockout of this gene are not responsive to leptin signaling and have decreased expression of, the α-MSH precursor, pro-opiomelanocortin [68].

### Molecular signatures in peripheral tissues suggest that metabolic changes may be regulated by changes in molecular clocks

**Figure 4:**
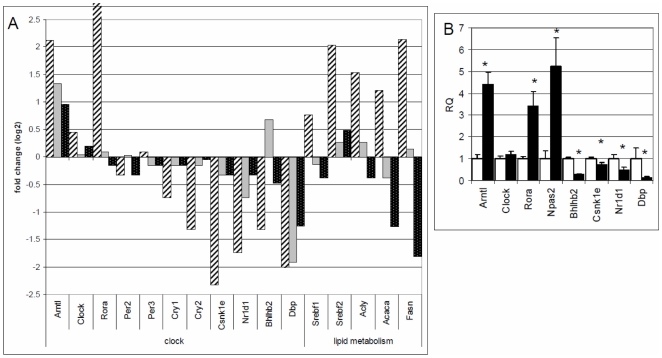
Changes in molecular signatures of circadian clocks genes and genes that regulate fatty acid synthesis during the transition from pregnancy to lactation. A) Gene expression fold changes from pregnancy day 20 (P20) to lactation day 1 (L1) in core clock genes and genes involved in fatty acid synthesis in mammary (striped bars), liver (gray bars) and adipose (dotted bars). Gene expression was measured using Rat 230 2.0 Affymetrix GeneChips and a linear model in which tissue type was the fixed effect was used to identify genes that were uniquely up and down-regulated in each tissue. Values are log base 2 fold change. B) Relative gene expression (RQ) of core clock genes was also measured in mammary tissue on P20 (white bars) and L1 (gray bars). Values are mean RQ (*n* = 5) ± SEM; * indicates a significant difference in gene expression on L1 relative to P20.

Our data show that multiple pathways and gene sets related to energy homeostasis are changed in peripheral tissues at the onset of lactation. Molecular signatures common to all the three tissues showed enrichment of gene sets associated with reception, integration and response to environmental and internal stimuli that are normally associated with the central nervous system. Transcriptomes of all three tissues also showed changes in molecular clock genes during the transition from pregnancy to lactation (Figure 4; Supplemental Table S10).

Circadian rhythms coordinate endogenous processes and circadian clocks are synchronized (entrained) to the external world, principally via light-dark cycles. Synchronization of circadian clocks to the external world enables organisms to anticipate and prepare for periodic and seasonal changes in their environment [69].

Daily and seasonal rhythms are coordinated in mammals by the master clock that lies in the suprachiasmatic nuclei (SCN) of the hypothalamus. Internal and external synchronizing factors affect the autoregulatory transcription–translation feedback loop of core clock genes that generate circadian rhythms [70]. Molecular clocks are also distributed in every organ and perhaps in every cell of the organism [71]–[74]. These tissue clocks are synchronized by endocrine, autonomic and behavioral cues that are dependent on the SCN, and in turn they drive the circadian expression of local transcriptomes, thereby coordinating metabolism and physiology of the entire organism.

Intracellular circadian rhythm generation occurs through an auto regulatory transcription–translation feedback loop [70]. The positive loop consists of ARNTL (aka BMAL1) and CLOCK gene products (and NPAS2 outside of the SCN), and the negative loop consists of the PER and CRY gene products [75]–[78]. ARNTL forms heterodimers with CLOCK and NPAS2; these complexes function as transcription factors that drive rhythmic expression of numerous output genes including their own repressors, PERS and CRYS [63], [64]. ARNTL expression is also regulated by Rev-erbα (NR1D1) and Rorα (RORA) that respectively repress or activate ARNTL transcription [79], [80]. The genes that RORA and NR1D1 regulate are often coordinately regulated by these two molecules, and crosstalk between RORA and NR1D1 likely acts to fine-tune their target physiologic networks, such as circadian rhythms, metabolic homeostasis, and inflammation [81]. Additionally, the basic helix loop helix transcription factors BHLH2 and BHLH3, aka Dec1 and Dec2, repress Clock-Arntl promoter activation [82]. CSNK1E, casein kinase 1, epsilon also acts as a negative regulator of circadian rhythmicity by phosphorylating PER1 and PER2 [83].

During the transition from pregnancy to lactation there was a significant (P<0.05) induction of ARNTL (4 fold), CLOCK (1.4 fold), NPAS2 (5 fold) and RORA (3 fold) in the mammary gland (Figure 4). Although an important clock gene, NPAS2, was not well measured on the gene expression arrays [84], we determined the expression levels of this gene by qPCR (Figure 4B). A significant decrease in expression of genes that generate the negative limb of circadian rhythms occurred in PER1, CRY1, NR1D1, BHLHB2 and CSNK1E, respectively, by 40%, 60%, 60%, 70% and 80% during the transition from pregnancy to lactation in the mammary gland. Significant expression changes in ARNTL, RORA, NR1D1, BHLHB2, CSNK1E and DBP during the pregnancy to lactation transition were confirmed and validated for mammary using qPCR (Figure 4B). It is important to note that since we collected the tissues at the same time of day on P20 and L1, these differences in gene expression are not due to sampling times; rather, differences are indicative of changes in amplitudes and/or patterns of genes that show circadian rhythms.

When expression statistics of core clock genes were examined for common up or down regulation during the transition from pregnancy to lactation, these data suggested that ARNTL, CLOCK and RORA genes were significantly induced, and expression of BHLHB2 and NR1D1 were significantly reduced in all three tissues (adjusted P<0.05; across the 15 arrays on P20 and L1; Supplemental Table S10). However when changes in expression of genes were examined within liver and adipose, only ARNTL was found to be significantly induced in the liver (1.3 fold). The fact that subtle changes in expression in core clock genes can only be picked up when arrays are examined across all three tissues may be due to the fact that the majority of transcriptome changes in these tissues occurs in an earlier phase of reproduction. The intimate interaction of metabolism and circadian clocks in peripheral tissues, suggests that the subtle changes evident in transcriptomes picked up when examined across the three tissues have a real biological significance. Further, the fact that the dam switches to a “catabolic condition” in late pregnancy to support rapid fetal growth [34], [85], which is geared up with the onset of lactation, suggests that in order to capture a window of large transcriptional changes in circadian clock and metabolic genes in liver and adipose we would need to compare non-pregnant and/or early pregnant animals with late pregnant and/or lactating animals.

Thus in general our data showed an induction of expression of the positive limb core clock genes and a suppression of expression of the negative limb of core clock genes. The transcriptional signature of the molecular clock suggests that the basal level of output genes that show a circadian rhythm of expression may be up-regulated at the onset of lactation, particularly in the mammary gland. Global temporal expression profiles of tissues, including liver, adipose, heart and SCN showed that a significant portion of the genome is under circadian control (in mammals, approximately 3–10% of all detectably expressed transcripts) [86]–[90]. Tissue-specific clock-controlled genes were found to be involved in rate-limiting steps of processes critical to the function of the organ. For example, in the liver coordinated circadian expression of genes encoding components of sugar, lipid, cholesterol and xenobiotic metabolic pathways were reported [86]–[88]. Transcription factors and enzymes involved in fatty acid synthesis including SREBF1, acetyl-CoA carboxylase (ACACA), fatty acid synthase (FASN) have also been reported to show circadian patterns of expression [91], [92]. Thus it is plausible that a circadian clock in mammary gland controls a unique set of genes important for its major function, lactation.

We examined the expression changes in genes involved in fatty acid synthesis during the transition from pregnancy to lactation in all three tissues in relation to changes in core clock genes (Figure 4A). The fatty acid synthesis genes (SREBF1, ACYL, ACACA, FASN) were selected based on their importance in milk fat synthesis and the fact that these genes respond to circadian entrainment [92]–[94]. The induction of core clock genes ARNTL, CLOCK, NPAS2, and RORA corresponded to the up regulation of genes that regulate fatty acid synthesis in mammary tissue during the transition from pregnancy to lactation. Significant changes in fatty acid synthesis genes were confirmed and validated in mammary tissue using qPCR (data not shown). It is interesting to speculate that the up regulation in expression of genes that regulate fatty acid synthesis and that have been shown to have diurnal patterns of expression are due to changes in molecular clocks at the onset of lactation in the mammary gland.

Although we only examined one time point across 2 days, others have shown that there are amplitude changes in core circadian clock genes in the mammary gland during the transition from pregnancy to lactation. Specifically, in mouse dams there is an increase in the amplitude of expression of Bmal1 (Arntl) and decrease in amplitude of expression of Per2 during the transition from pregnancy to lactation [95]–[97]. Preliminary work in our lab supports that mammary tissue in fact possesses a functional clock that can be reset by external signals. We tested the ability of a mammary epithelial cell line, MAC-T, to be synchronized in culture by serum treatment. Our studies showed that treating mammary epithelial cell cultures with serum for 2 hrs initiated a circadian pattern of expression of *Bmal1* (ARNTL) as measured with qPCR every 4 hrs for 48 hrs.

Interestingly, homozygous *Clock* mutant mice, which have a genetic mutation that disrupts circadian rhythms, exhibit severe alterations in energy balance, with a phenotype associated with metabolic syndrome, including obesity, hyperlipidemia, hepatic steatosis, high circulating glucose, and low circulating insulin [99]. Offspring of these *Clock* mutant mice fail to thrive, suggesting that their milk production may not be adequate enough to nourish their young [100], [101]. The effect of circadian clocks on milk production is evident in both the diurnal variation in milk composition [102], [103], [104] as well as the photoperiod effect on milk quality and quantity in cattle and other ruminants [105]–[117]. These studies have shown that altering the photoperiod in cows influences milk production and composition and results in changes in circulating levels of hormones known to be important for milk production.

**Figure 5:**
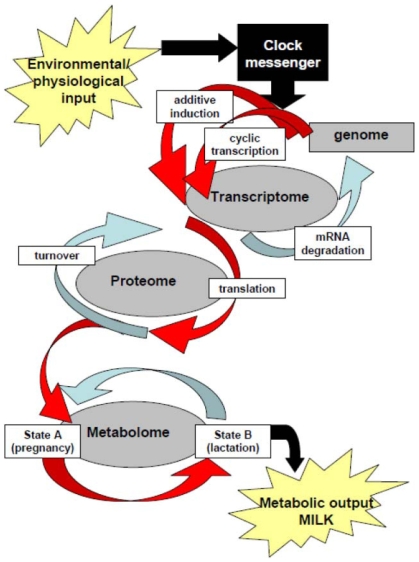
Schematic of how molecular clocks affect metabolic output, as modified from [126]. The master clock receives input from the external environment as well as the mammals physiological state and these factors affect the autoregulatory transcription–translation feedback loop of core clock genes that generate circadian rhythms [70]. Molecular clocks in peripheral tissues are synchronized by endocrine, autonomic and behavioral cues that are dependent on the master clock, and in turn they drive the circadian expression of local transcriptomes, thereby coordinating metabolism and physiology of the entire organism. We envision that during the transition from pregnancy to lactation the master clock is modified by environemental and physiological cues that it receives. In turn the master clocks coordinates changes in endocrine milieu and autonomic nervous systems that send signals to peripheral tissues. These signals stimulate the induction of expression of the positive limb core clock genes and suppression of expression of the negative limb of core clock genes in mammary, liver and adipose tissues, and result in up regulation of genes that show circadian patterns of expression. These changes are needed to accommodate for the increased metabolic demands of milk synthesis and to stimulate copious milk production.

We hypothesize that the master clock modifies peripheral clocks and hormonal levels at the onset of lactation in order to coordinate the changes needed to stimulate lactogenesis and accommodate the increased metabolic demands of milk synthesis. Following modification of the clocks there is a change in the mammal's metabolome that results in the partitioning of nutrients to the mammary which in turn are used to synthesize milk (Figure 5). Based on this hypothesis we believe that environmental inputs and physiological inputs received through the master clock in the suprachiasmatic nucleus (day light, food availability, metabolic stores, social cues, stress, etc.) can profoundly influence milk production and composition.

### Conclusion

Multiple pathways and gene sets related to energy homeostasis are changed in mammary, liver and adipose tissues during the transition from pregnancy to lactation. Gene sets enriched with genes up regulated during the pregnancy to lactation transition in the mammary gland reflect the turning on of secretory processes in this tissue and the down regulation of catabolic processes. Gene sets enriched with genes up regulated in liver were related to P450 pathways which catalyze many reactions involved in drug metabolism and synthesis of cholesterol, steroids and other lipids, and the transcriptional signature of genes down regulated in liver at the onset of lactation suggest that there is a reduction in breakdown fatty acids, so that fats can be spared for milk synthesis in the mammary gland. There was a similarity between the molecular signature of adipose tissue at the onset of lactation and adipose tissue from rats exposed to long-term caloric restriction, in particular the enrichment of up regulated genes in inflammation related genes sets and the enrichment of down regulated genes in cytoskeletal and ECM gene sets. The majority of the gene sets enriched with genes commonly up regulated among all three tissues were related to metabolic processes. Genes commonly down regulated among the three tissues were related to perception and transduction of external stimuli, light and taste as well as rhythmic processes.

Molecular signatures of mammary, liver and adipose were also enriched with gene sets classically associated with central nervous systems reception, integration and response to environmental and internal stimuli. In particular we found that core clock genes were commonly changed among the three tissues at the onset of lactation. These signatures illustrate the complexity of homeorhetic adaptations as well as the role of the nervous system in orchestrating the response, and suggested that changes in multiple tissues may be coordinated by changes in molecular clocks. We envision that environmental gene interactions leading to taxonomic variation in milk composition are mediated through changes in molecular clocks, which in turn mediate changes in the animal's transcriptome, proteome and metabolome, and thus metabolic output, milk.

## Materials and Methods

### Animals and treatment conditions

Ten time-bred female Sprague-Dawley (190–280 g) rats (Taconic Farms, Germantown, NY) were obtained on day 2 of pregnancy (P2) and were used according to a protocol approved by the NASA Animal Care Committee. During the period of pregnancy (P2) to post natal day 1, the dams were individually housed in maternity cages and maintained under standard colony conditions (12:12 light/dark cycle [0600∶1800]; 21+/− 1°C at 30–50% humidity). Standard rat chow (Purina #5012 pellets) and water were available *ad libitum* during the experimental period. On P20, five rats were removed from the maternity cages, anaesthetized with isoflurane, and tissues were collected for analysis. Animals were euthanized by cardiac puncture. Real time videography was used to identify the precise time of birth so that dams could be euthanized within 18–36 hrs after delivery of the pups (L1). All dams were euthanized for tissue collection between Zeitgeber time (ZT) 09:00–12:00 on P20 (mean ZT 10:45) and ZT 11:00–13:00 on L1 (mean ZT 12:17), with ZT 00:00 =  time of lights on.

### Dam tissue collection and metabolic assays

On day P20 and L1, mammary glands, liver and visceral adipose tissue samples were collected from anesthetized dams. Mammary and liver tissues were kept in 25 mmol/L Tris, 0.25 mol/L sucrose, 1 mmol/L EDTA (pH 7.3) on ice, and adipose tissue was kept in saline at 37°C to maintain tissue viability during the time between tissue removal and incubation for metabolic analysis. As described previously [118], the *in-vitro* rate of incorporation of ^14^C-labeled glucose into lipids was used as an indicator of the *in-vivo* metabolic capacity of mammary, liver and adipose tissue on P20 and L1 [119], [120]. Glucose incorporation into lipids was calculated and expressed as nmoles of glucose incorporated per 100 mg tissue per 1 h of incubation.

### Isolation of total RNA

At each experimental time point, mammary gland #4, liver and visceral adipose tissues were collected from anesthetized dams and were snap frozen in liquid nitrogen and stored at −80°C. Total RNA was extracted from frozen liver and mammary tissue using Trizol® Reagent (Invitrogen, Carlsbad, CA) according to manufacturer's instructions. Total RNA was extracted from visceral adipose tissue using RNeasy Lipid Tissue Mini Kit (QIAGEN Inc., Valencia, CA) as detailed by the manufacturer. Isolated RNA was resuspended in nuclease free water (Ambion, Austin, TX), and quantity and quality of the RNA was assessed using the Nanodrop® ND-1000 UV-Vis Spectrophotometer (Nanodrop Technologies, Wilmington, DE) and on the Nanochip using the Bioanalyzer 2100 (Agilent Inc., Palo Alto, CA), respectively. RNA integrity number (RIN) for all samples was ≥7.0.

### RNA preparation for microarrays

Total RNA from all 3 tissues of 5 rat dams on P20 and 5 rat dams on L1 was amplified and biotinylated using NuGEN's Ovation Biotin System (NuGEN, San Carlos, CA) to generate products for the Rat 230 2.0 GeneChips (Affymetrix, Santa Clara, CA). Gene expression data were generated following the manufacturer supplied protocols. Briefly, GeneChips were hybridized in an Affymetrix 640 Hybridization oven at 45°C for 16 hours with 60 rpm rotation. After hybridization, gene chips were washed on a Fluidics station (Affymetrix, Santa Clara, CA), stained, and then scanned using an Affymetrix Genearray scanner GSC3000, with 7G upgrade (Affymetrix). The efficiency of amplification and hybridization were assessed by incorporating Affymetrix Poly-A RNA and Hybridization controls with every sample. The microarray data were deposited in the Gene Expression Omnibus (GEO; www.ncbi.nlm.nih.gov/geo, accession no. GSE12132).

### Microarray gene expression analysis

Gene expression analysis was performed using BioConductor version 2.0 software [121], and normalized probe set statistics were calculated using the RMA statistic as implemented in the BioConductor package using updated probeset mappings such that a single probeset describes each well measured gene [84], [122], [123]. Two types of gene expression analysis were performed. Within each tissue type group (mammary, liver, adipose), L1 (n = 5) and P20 (n = 5) samples were compared using a two-sample comparison of means. For example, for each gene, the expression values derived from the mammary L1 samples were compared to the expression values derived from mammary P20 samples. Individual gene expression differences were evaluated using a moderated t-test as implemented in the LIMMA package [30]. The corresponding nominal P-values were adjusted to control for multiple testing using the false discovery rate method. In addition, for each tissue type (mammary, liver, adipose) the median gene expression value of the P20 samples was subtracted from the gene expression value in each L1 sample. For example, for each gene, the median expression value of the P20 mammary samples was subtracted from the expression value in each L1 mammary sample. Individual genes that were commonly up-regulated and down-regulated across all L1 tissues were identified using a derivative of a one sample test of location as implemented in the LIMMA package [30]. The corresponding nominal P-values were adjusted to control for multiple testing using the false discovery rate method.

For gene ontology analysis, genes were grouped into sets based on Gene Ontologies (GO). This was performed by converting human GO sets to corresponding rat GO sets using NCBI homologene. For each gene in a given GO set, the expression of the gene in each L1 sample was compared to the median expression of gene in the P20 samples. The entire set of genes in each ontology was given an enrichment score using the parametric gene set enrichment analysis method (PGSEA) to test for enrichment in up or down-regulated genes [39], [40]. Ontologies were scored only if they contained at least 10 genes. A derivative of a one sample test of location as implemented in the LIMMA package was performed on the resulting enrichment scores to identify pathways that were consistently up or down regulated in each tissue type and across all tissue types [30]. The corresponding nominal P-values were adjusted to control for multiple testing using the false discovery rate method.

### Quantitative polymerase chain reaction (QPCR)

We used MIQE guidelines when measuring gene expression with QPCR [124]. Briefly, total RNA was extracted from mammary tissue using Trizol® Reagent, as above, and repurified using the QIAGEN Rneasy kit with DNase treatment (QIAGEN Inc.) according to manufacturer. Quantity and quality of RNA were determined as described above, and RIN were ≥8.0. Equivalent amounts of total RNA (1 µg) from each tissue sample (P20 n = 5 and L1 n = 5) was reverse transcribed into cDNA (Applied Biosystems) according to manufacturer's instructions. Oneµl of cDNA was used per well for qPCR. qPCR analysis was performed using the ABI Prism 7500 (Applied Biosystems, Foster City, CA) and a unique TaqMan® Assays-on-Demand^TM^ Gene Expression kit (AOD; Applied Biosystems) specific for rat. Samples, no template controls (NTC) and no reverse transcription controls (NoRT) and TaqMan reaction mixes (20 µl) were loaded into MicroAmp Fast Optical 96-well reaction plates and sealed with MicoAmp Optical adhesive film (Applied Biosystems).

Three reference genes were tested to compare efficiency of amplification with target genes: β_2_Microglobulin (B2M, Assay ID Rn00560865_m1); Actin, beta (Actb, Assay ID Rn00667869_m1); ribosomal protein L10A (Rpl10a, Assay ID Rn00821239_g1). Target gene TaqMan hydrolysis probes were as follows: aryl hydrocarbon receptor nuclear translocator-like (Arntl, Assay ID Rn00577590_m1); circadian locomoter output cycles kaput (Clock, Assay ID Rn00573120_m1); neuronal PAS domain protein 2 (NPAS2, Assay ID Rn01438224_m1); RAR-related orphan receptor alpha (RORA, Assay ID Rn01173769_m1); basic helix-loop-helix domain containing, class B2 (Bhlhb2, Assay ID Rn00584155_m1); casein kinase 1, epsilon (Cskn1e, Assay ID Rn00581130_m1); nuclear receptor subfamily 1, group D, member 1 (NR1D1 Assay ID Rn00595671_m1); sterol regulatory element binding protein-1 (Srebp1, Assay ID Rn01495759_m1); acetyl CoA carboxylase-α (Acaca, Assay ID Rn00573474_m1); and fatty acid synthase (Fasn, Assay ID Rn005569117_m1). The qPCR data across the tissues was normalized relative to the abundance of a validated endogenous control [β_2_Microglobulin, (B2M, Assay ID Rn00560865_m1)] mRNA.

The variation of quantification cycle (C_q_) across samples was compared among three reference genes (B2M, Actb, Rpl10a) to select the most appropriate reference gene for our study. The range of C_q_ was calculated across all samples for each reference gene and differences of C_q_ among all samples were ≤1.7 for Actb and B2M, and ≤2 for Rpl10a. Thus Actb and B2M were used as the reference genes for the study. Secondly PCR amplification efficiency was compared between the Actb reference gene and each of the target genes using calibration curves. The slope of Actb calibration curve was −3.2. Slopes of target gene calibration curves ranged from −3.1 to −3.4, these data indicate that the amplification efficiency is similar enough (<0.25 difference in slope from reference gene) between target and reference genes to use the delta delta C_q_ (Δ Δ C_q_) method for calculating differences in relative gene expression (RQ). The mean C_q_ of target and mean C_q_ of reference gene for each sample were calculated from duplicate wells. The relative amounts of target gene expression for each sample were then calculated using the formula 2^−ΔΔCq^ [125]. Differences in gene expression were calculated using a student's T-test (http://www.physics.csbsju.edu/stats/t-test_NROW_form.html) and data were presented as mean +/− SEM.

## Supporting Information

Figure S1 A) Gene ontology and B) KEGG Pathway gene sets enriched with up regulated genes or down regulated genes in mammary during the transition from pregnancy to lactation. Each column represents data from an individual lactating (L1) rat compared to the average of the 5 pregnant rats (P20). For each L1 rat comparison, enrichment scores for each pathway were calculated and the pathways that were most consistently deregulated across the tissues were identified and the results plotted as a heat map [30]. Red indicates an enrichment of up regulated genes in the ontology/pathway and blue indicates enrichment of down regulated genes in the ontology/pathway during the P20 to L1 transition. Ontologies/Pathways were only scored if they had at least 10 genes represented in each category.
(1.02 MB TIF)

Figure S2 A) Gene ontology and B) KEGG Pathway gene sets enriched with up regulated genes or down regulated genes in liver during the transition from pregnancy to lactation. Each column represents data from an individual lactating (L1) rat compared to the average of the 5 pregnant rats (P20). For each L1 rat comparison, enrichment scores for each pathway were calculated and the pathways that were most consistently deregulated across the tissues were identified and the results plotted as a heat map [30]. Red indicates an enrichment of up regulated genes in the ontology/pathway and blue indicates enrichment of down regulated genes in the ontology/pathway during the P20 to L1 transition. Ontologies/Pathways were only scored if they had at least 10 genes represented in each category.
(1.02 MB TIF)

Figure S3 A) Gene ontology and B) KEGG Pathway gene sets enriched with up regulated genes or down regulated genes in adipose during the transition from pregnancy to lactation. Each column represents data from an individual lactating (L1) rat compared to the average of the 5 pregnant rats (P20). For each L1 rat comparison, enrichment scores for each pathway were calculated and the pathways that were most consistently deregulated across the tissues were identified and the results plotted as a heat map [30]. Red indicates an enrichment of up regulated genes in the ontology/pathway and blue indicates enrichment of down regulated genes in the ontology/pathway during the P20 to L1 transition. Ontologies/Pathways were only scored if they had at least 10 genes represented in each category.
(0.86 MB TIF)

Table S1 Expression changes in genes commonly up and down regulated across all three tissues (adjusted P<0.001; common) during the transition from pregnancy to lactation, and changes within mammary, liver and adipose tissues. Values are log base 2 fold change and corresponding adjusted and unadjusted (nominal) p-values.
(0.03 MB XLS)

Table S2 Changes in expression of genes that enrich the KEGG_PATHWAY:hsa03320:PPAR signaling pathway, selected based on enrichment by 22 genes that were down regulated in mammary (p<0.05, unadjusted) during transition from pregnancy to lactation. Values are log base 2 fold change, adjusted and unadjusted (nominal) p-values as calculated across all three tissues (common) and within mammary, liver and adipose tissue.
(0.02 MB XLS)

Table S3 Changes in expression of genes that enrich the KEGG_PATHWAY:hsa04920:Adipocytokine signaling pathway, selected based on enrichment by 8 genes that were down regulated in liver (p<0.05, unadjusted) during transition from pregnancy to lactation. Values are log base 2 fold change, adjusted and unadjusted (nominal) p-values as calculated across all three tissues (common) and within mammary, liver and adipose tissue.
(0.02 MB XLS)

Table S4 Changes in expression of genes that enrich the GOTERM_MF_ALL:GO:0004888∼transmembrane receptor activity, selected based on enrichment by 33 genes that were down regulated in liver (p<0.05, unadjusted) during transition from pregnancy to lactation. Values are log base 2 fold change, adjusted and unadjusted (nominal) p-values as calculated across all three tissues (common) and within mammary, liver and adipose tissue.
(0.02 MB XLS)

Table S5 Changes in expression of genes that enrich the GOTERM_CC_ALL:GO:0043292∼contractile fiber, selected based on enrichment by 12 genes that were down regulated in adipose (p<0.05, unadjusted) during transition from pregnancy to lactation. Values are log base 2 fold change, adjusted and unadjusted (nominal) p-values as calculated across all three tissues (common) and within mammary, liver and adipose tissue.
(0.02 MB DOC)

Table S6 Changes in expression of genes that enrich the GOTERM_MF_ALL:GO:0003700∼transcription factor activity, selected based on enrichment by 112 genes that were commonly up regulated in all three tissues (p<0.05, adjusted) during transition from pregnancy to lactation. Values are log base 2 fold change, adjusted and unadjusted (nominal) p-values as calculated across all three tissues (common) and within mammary, liver and adipose tissue.
(0.05 MB XLS)

Table S7 Changes in expression of genes that enrich the GOTERM_MF_ALL:GO:0003700∼transcription factor activity, selected based on enrichment by 97 genes that were commonly down regulated in all three tissues (p<0.05, unadjusted) during transition from pregnancy to lactation. Values are log base 2 fold change, adjusted and unadjusted (nominal) p-values as calculated across all three tissues (common) and within mammary, liver and adipose tissue.
(0.04 MB XLS)

Table S8 Changes in expression of genes that enrich the GOTERM_BP_ALL:GO:0048511∼rhythmic process, selected based on enrichment by 13 genes that were commonly down regulated in all three tissues (p<0.05, unadjusted) during transition from pregnancy to lactation. Values are log base 2 fold change, adjusted and unadjusted (nominal) p-values as calculated across all three tissues (common) and within mammary, liver and adipose tissue.
(0.02 MB XLS)

Table S9 Changes in expression of genes that enrich the GOTERM_BP_ALL:GO:0050953∼sensory perception of light stimulus, selected based on enrichment by 24 genes that were commonly down regulated in all three tissues (p<0.05, unadjusted) during transition from pregnancy to lactation. Values are log base 2 fold change, adjusted and unadjusted (nominal) p-values as calculated across all three tissues (common) and within mammary, liver and adipose tissue.
(0.02 MB XLS)

Table S10 Changes in core clock gene set during the transition from pregnancy to lactation across the three tissues (common) and within mammary, liver and adipose tissue. Values are log base 2 fold change and corresponding adjusted and unadjusted (nominal) p-values.
(0.02 MB XLS)
