# Supplementary figures and images for: Molecular Signatures Reveal Circadian Clocks May Orchestrate the Homeorhetic Response to Lactation
Source: PLoS One. 2009 Oct 9;4(10):e7395. doi: 10.1371/journal.pone.0007395 (PMC2754660; doi:10.1371/journal.pone.0007395)

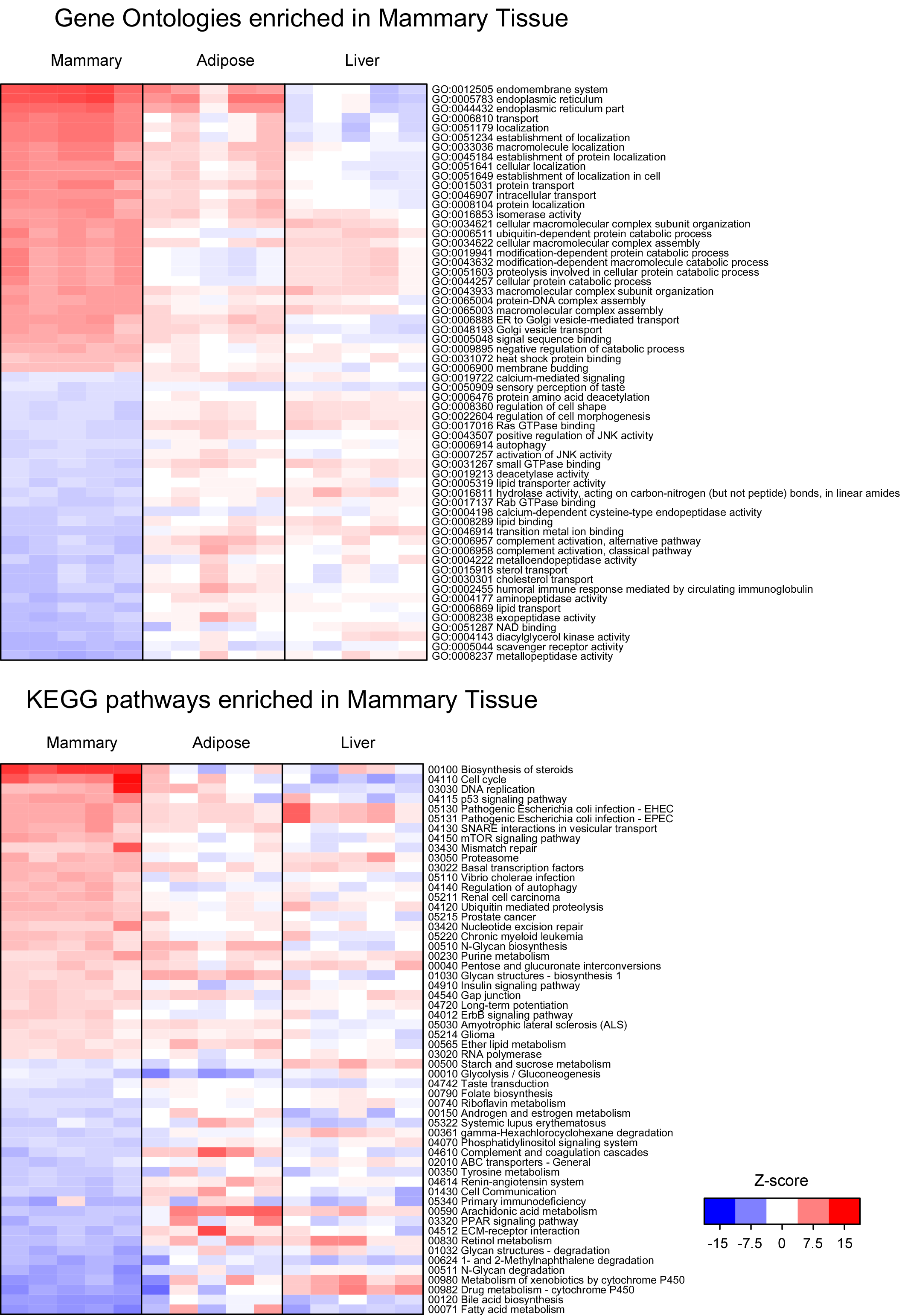

Supplement: Figure S1 — A) Gene ontology and B) KEGG Pathway gene sets enriched with up regulated genes or down regulated genes in mammary during the transition from pregnancy to lactation. Each column represents data from an individual lactating (L1) rat compared to the average of the 5 pregnant rats (P20). For each L1 rat comparison, enrichment scores for each pathway were calculated and the pathways that were most consistently deregulated across the tissues were identified and the results plotted as a heat map [30]. Red indicates an enrichment of up regulated genes in the ontology/pathway and blue indicates enrichment of down regulated genes in the ontology/pathway during the P20 to L1 transition. Ontologies/Pathways were only scored if they had at least 10 genes represented in each category. (1.02 MB TIF) [file pone.0007395.s001.tif]

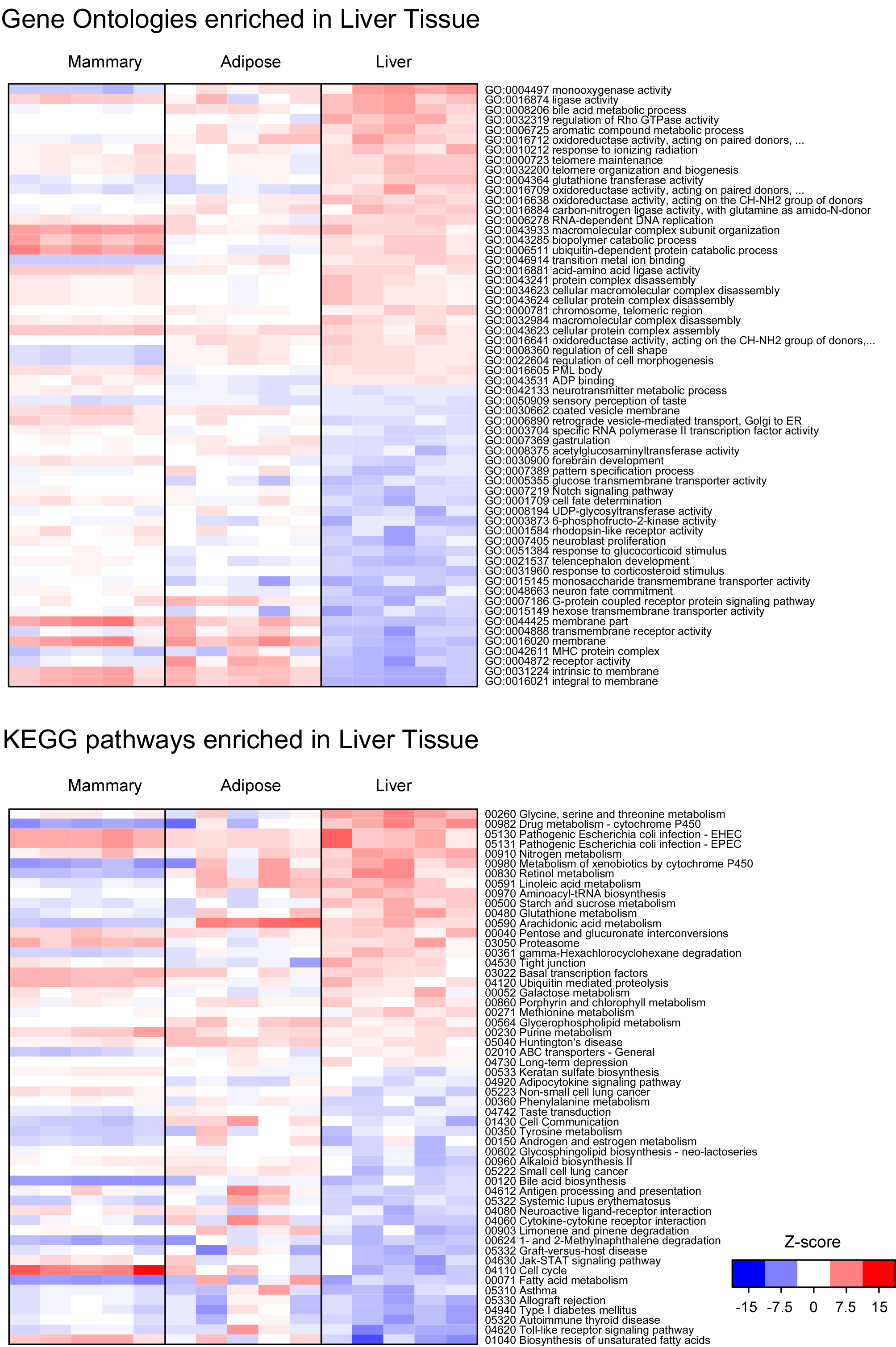

Supplement: Figure S2 — A) Gene ontology and B) KEGG Pathway gene sets enriched with up regulated genes or down regulated genes in liver during the transition from pregnancy to lactation. Each column represents data from an individual lactating (L1) rat compared to the average of the 5 pregnant rats (P20). For each L1 rat comparison, enrichment scores for each pathway were calculated and the pathways that were most consistently deregulated across the tissues were identified and the results plotted as a heat map [30]. Red indicates an enrichment of up regulated genes in the ontology/pathway and blue indicates enrichment of down regulated genes in the ontology/pathway during the P20 to L1 transition. Ontologies/Pathways were only scored if they had at least 10 genes represented in each category. (1.02 MB TIF) [file pone.0007395.s002.tif]

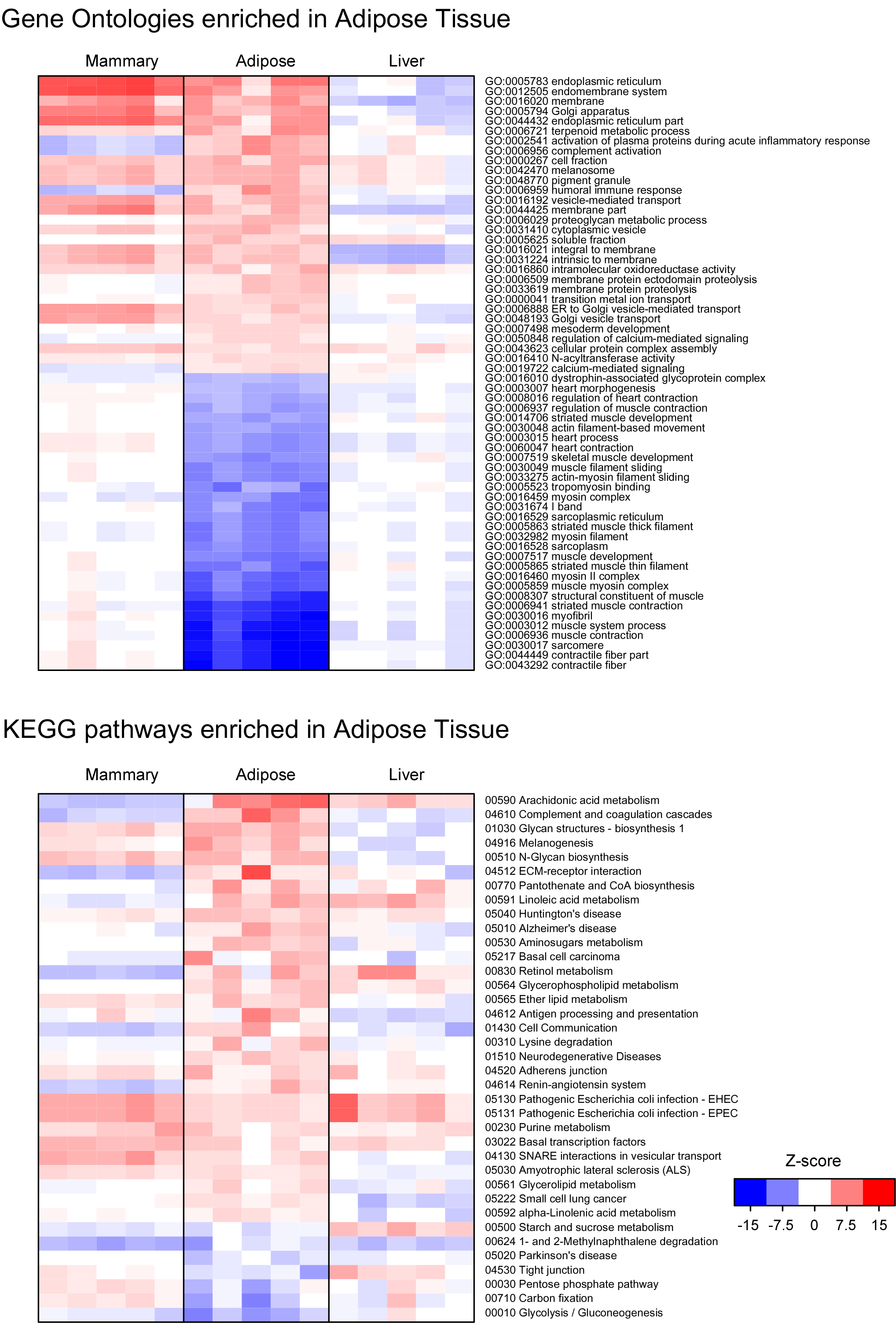

Supplement: Figure S3 — A) Gene ontology and B) KEGG Pathway gene sets enriched with up regulated genes or down regulated genes in adipose during the transition from pregnancy to lactation. Each column represents data from an individual lactating (L1) rat compared to the average of the 5 pregnant rats (P20). For each L1 rat comparison, enrichment scores for each pathway were calculated and the pathways that were most consistently deregulated across the tissues were identified and the results plotted as a heat map [30]. Red indicates an enrichment of up regulated genes in the ontology/pathway and blue indicates enrichment of down regulated genes in the ontology/pathway during the P20 to L1 transition. Ontologies/Pathways were only scored if they had at least 10 genes represented in each category. (0.86 MB TIF) [file pone.0007395.s003.tif]
