# Supplementary material for: Molecular Signatures Reveal Circadian Clocks May Orchestrate the Homeorhetic Response to Lactation
Source: PLoS One. 2009 Oct 9;4(10):e7395. doi: 10.1371/journal.pone.0007395 (PMC2754660; doi:10.1371/journal.pone.0007395)
Supplement: Supplementary file 2 [file pone.0007395.s008.doc]

## Sheet: Sheet1

Table S5.  Changes in expression of genes that enrich the GOTERM_CC_ALL:GO:0043292~contractile fiber, selected based on enrichment by 12 genes that were down regulated in adipose (p<0.05, unadjusted) during transition from pregnancy to lactation.  Values are log base 2 fold change, adjusted and unadjusted (nominal) p-values as calculated across all three tissues (common) and within mammary, liver and adipose tissue.
		Common			Mammary			liver			adipose
entrez	symbol	fold_change	adjusted_p	unadjusted_p	fold_change	adjusted_p	unadjusted_p	fold_change	adjusted_p	unadjusted_p	fold_change	adjusted_p	unadjusted_p
308709	Dmn	-0.378883998616183	0.0318494871070834	0.00635538031577687	0.041281250313963	0.94777333063776	0.902134297910254	-0.26183334134451	0.904987657262004	0.437906687061697	-0.916099904818005	0.357433078480513	0.0102880038319551
29437	Acta1	-0.789131875757077	0.100993630053996	0.0409710513098307	0.44700805050235	0.795105377199009	0.671559658369363	-0.039019372853951	0.99746381451828	0.9704272402785	-2.77538430491963	0.377712140237498	0.0128272147938544
64353	Pdlim5	-0.238291680038436	0.037760911167576	0.00850988636838043	-0.165337959547223	0.471393519708526	0.3134003416035	-0.0522681360658925	0.981142750695185	0.747918086081459	-0.497268944502193	0.268483215502796	0.00454921321997892
140939	Trim63	-0.531802947179961	0.0290856046674537	0.00537187108147591	-0.00129682540257781	0.998895810789676	0.998355341692737	-0.1473863451888	0.98943197456134	0.814869006909381	-1.44672567094851	0.477070440556552	0.0279271136809959
24583	Myh3	-0.34927725240423	0.0131360769059517	0.00132458873712052	-0.186365588258424	0.744502056576273	0.605270931923078	-0.112477007967574	0.981414392967445	0.754727725544182	-0.748989160986695	0.52746691745403	0.0448765248868944
29275	Actc1	-0.373007765940625	0.090219759525775	0.0344630000384013	0.226522727811081	0.81869704989129	0.70327816067377	-0.0889118591588289	0.991068145455528	0.881021580516534	-1.25663416647413	0.524897378023841	0.0417630507882551
27064	Ankrd1	-0.417930572806001	0.0294395134840127	0.00555515723788362	-0.382457249983426	0.598703407655826	0.436853897660762	0.0320949812450326	0.992053666589214	0.892581175229253	-0.997375118211964	0.534533952017383	0.0491894536598443
316740	Myom1	-0.704823326278098	0.0099441298179183	0.000754914797176848	-0.641255755613656	0.489476655529184	0.329642324439456	0.0766934804863681	0.993189962559501	0.906385465199317	-1.54990770370701	0.461050375729702	0.0234651613714371
83708	Mybph	-0.489281102751978	0.0168675954930463	0.0020859492771572	-0.239039485611998	0.783419749247457	0.655769499288676	-0.0760678233572736	0.991874406724251	0.887013371240686	-1.15273599928666	0.514198755465361	0.038503010697463
24837	Tnnt2	-0.307746000834242	0.0271732480428519	0.00479428325553931	-0.220510999907825	0.563297114359453	0.399615255197358	0.0552612188891346	0.989634854115493	0.83180938228377	-0.757988221484034	0.296095382074486	0.00654895742310339
317258	Itgb1bp2	-0.376291267469094	0.0978804249677989	0.039095916869322	0.246951101850422	0.763011039132306	0.62903476961935	-0.106881567305291	0.989634854115493	0.834099929147968	-1.26894333695241	0.433498207893122	0.0182044520596373
25566	Tmod1	-0.320747962965415	0.0509962186214156	0.0139885104266391	-0.258058771753033	0.678602206263074	0.52490625202324	0.161681132689761	0.97329414336212	0.689712457495231	-0.865866249832974	0.520158047231446	0.0395410579631012

## Sheet: Sheet2

## Sheet: Sheet3
